# Case Report: Haploidentical Bone Marrow Transplantation in Two Brothers With Wiskott–Aldrich Syndrome Using Their Father as the Donor

**DOI:** 10.3389/fped.2021.647505

**Published:** 2021-10-27

**Authors:** Jasmine Smith, Jessica Hass Alfonso, Naresh Reddivalla, Pablo Angulo, Emmanuel Katsanis

**Affiliations:** ^1^Department of Pediatrics, University of Arizona, Tucson, AZ, United States; ^2^Banner University Medical Center, Tucson, AZ, United States; ^3^Banner Children's at Desert, Mesa, AZ, United States; ^4^Department of Immunobiology, University of Arizona, Tucson, AZ, United States; ^5^Department of Medicine, University of Arizona, Tucson, AZ, United States; ^6^Department of Pathology, University of Arizona, Tucson, AZ, United States; ^7^The University of Arizona Cancer Center, Tucson, AZ, United States

**Keywords:** Wiskott Aldrich syndrome (WAS), haploidentical hematopoietic stem cell transplantation, cyclophosphamide, myeloablative allogeneic hematopoietic cell transplantation, immunodeficiency

## Abstract

Wiskott–Aldrich syndrome (WAS) is an X-linked genetic disorder with a variable phenotypic expression that includes thrombocytopenia, eczema, and immunodeficiency. Some patients may also exhibit autoimmune manifestations. Patients with WAS are at increased risk of developing malignancies such as lymphoma. Allogeneic hematopoietic cell transplantation remains the only curative treatment. Haploidentical bone marrow transplantation (haplo-BMT) with post-transplant cyclophosphamide (PT-CY) has more recently been applied in WAS. Here, we report two brothers who underwent successful T-cell replete haplo-BMT with PT-CY at ages 9 months and 4 years using their father as the donor. Our myeloablative regimen was well-tolerated with minimal organ toxicity and no acute or chronic graft vs. host disease (GvHD). Haplo-BMT may be considered as a safe and effective option for patients with WAS who do not have available human leukocyte antigen (HLA) matched donors.

## Introduction

Wiskott–Aldrich syndrome (WAS) is an X-linked disorder characterized by the triad of microthrombocytopenia, eczema, and immunodeficiency, though all three are not required for diagnosis, as some patients may present with partial or variable phenotypic expression (1–3). Patients with WAS are also at increased risk for autoimmune manifestations as well as malignancies, especially lymphomas. WAS is caused by mutations in the WAS gene, which encodes the WAS protein involved in actin polymerization, cytoskeleton remodeling, signaling events, and the immunologic synapse (4–7). With the inappropriate functioning of the WAS gene, both T and B cells are affected, leading to severe immunodeficiency.

Allogeneic hematopoietic cell transplantation (HCT) remains the only curative treatment for WAS. Matched sibling donor (MSD) transplantation has resulted in event-free-survival > 90%, compared to 70–90% in matched unrelated donor (MUD) and umbilical cord blood (UCB) and 55–90% in mismatched unrelated donor transplantation (MMUD) (2, 8–11). With the increasing application of haploidentical bone marrow transplantation (haplo-BMT) with post-transplant cyclophosphamide (PT-CY) for hematologic malignancies, there have been a limited number of case reports and small case series in the last 3 years on its use in patients with WAS. Herein, we report two brothers with WAS who underwent successful T-cell replete haplo-BMT from their father using myeloablative conditioning (MAC) and PT-CY. The regimen was well-tolerated, resulting in complete donor engraftment and no acute or chronic graft vs. host disease (GvHD).

## Case Series

The older brother (patient #2) presented for his 1-year well child visit with eczema and purpura, and was found to have a platelet count of 5 × 10^9^/L. He was admitted for presumed immune thrombocytopenic purpura (ITP) and received intravenous immunoglobulin (IVIG) with a marginal response in his platelet count. Given the persistent eczema and thrombocytopenia with a low mean platelet volume, genetic testing was performed, which confirmed missense mutation, 291G>A, in the WAS gene (R86H). He was referred to our hematopoietic cell therapy and transplant program when he was 17 months of age for bone marrow (BM) transplant evaluation. No HLA-matched related or unrelated donor was identified. As his mother was pregnant, the parents indicated that they wished to wait in the event that the upcoming sibling may be a match. In the interim, the patient received monthly infusions of IVIG and IV pentamidine for *Pneumocystis jirovecii* prophylaxis while his platelet count ranged between 30 and 40 × 10^9^/L.

Patient #1 (younger brother) was born 2 years after his brother. While he proved to be an HLA match, he unfortunately had thrombocytopenia and genetic testing confirmed the same mutation in the WAS gene. In his first 7 months of life, his platelet count ranged between 50 and 80 × 10^9^/L, but he then developed transfusion refractory thrombocytopenia (1–5 × 10^9^/L), which did not respond to IVIG, steroids, or eltrombopag (12). He had no evidence of splenic sequestration and HLA antibody testing failed to reveal the presence of either class I or II antibodies. Because of his severe purpura, it was therefore decided to proceed to haplo-BMT with the younger sibling first.

**Figure 1:**
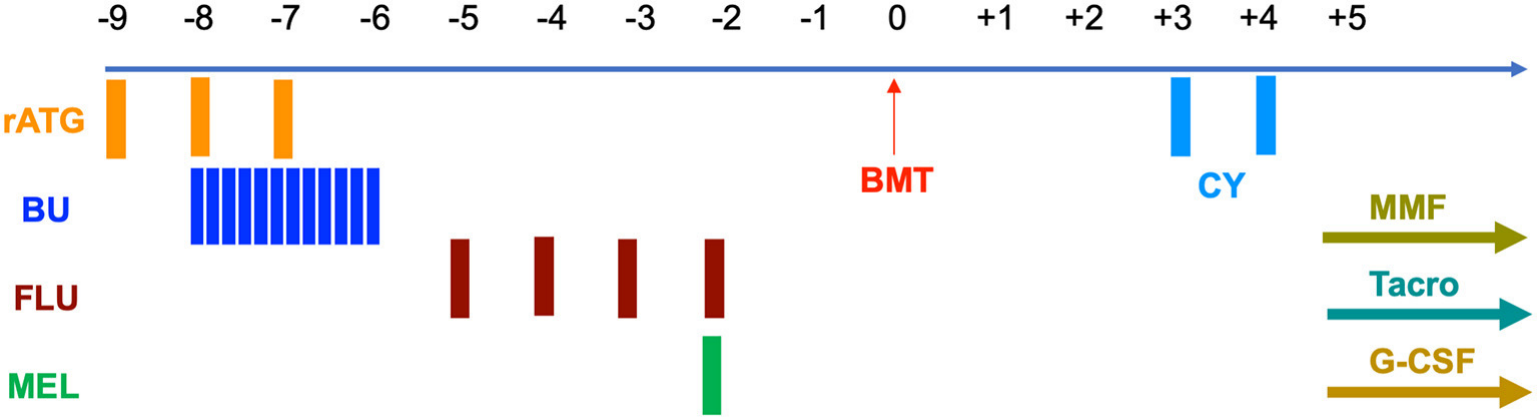
Conditioning regimen and GvHD prophylaxis.

Prior to transplantation, both patients underwent testing for donor specific anti-HLA antibodies that were negative. Conditioning was myeloablative and consisted of rabbit anti-thymocyte globulin 4.5 mg/kg IV over days −9 to −7, busulfan (BU) 1 mg/kg IV q 6 h for 12 doses on days −8 to −6, fludarabine (FLU) 30 mg/m^2^ IV on days −5 to −2, and melphalan (MEL) 100 mg/m^2^ IV on day −2 (Figure 1). Busulfan pharmacokinetics were performed after the first dose with an estimated area under the curve (AUC) average exposure of 4.44 mg × h/L per dose for the younger sibling and 4.11 for the older (Table 1). BM stem cells were freshly harvested from their father in both cases. GVHD prophylaxis included PT-CY 50 mg/kg IV on days +3 and +4 followed by initiation of mycophenolate mofetil (MMF) and tacrolimus on day +5. In the absence of GvHD, MMF was discontinued on day +28 without taper and tacrolimus was weaned beginning on day +100 over the next 3 months.

**Table 1:** Patient and transplant characteristics.

Gender	M	M
Age at BMT (yr)	0.7	4.0
Conditioning	ATG-BFM	ATG-BFM
BU AUC (mg × hr/L)	4.44	4.11
BMT donor	Father	Father
HLA match	5/10	5/10
DSA	no	no
Donor blood type	A+	A+
Recipient blood type	O+	AB+
CD34^+^ cells × 10^6^/kg	5.41	5.12
CD45^+^ cells × 10^8^/kg	3.65	4.17
CD3^+^ cells × 10^6^/kg	n/d	0.6
GvHD prophylaxis (days post-BMT)
PT-CY	+3, +4	+3, +4
MMF	+5 to +28	+5 to +28
Tacrolimus	+5 to +159	+5 to +172
G-CSF	+	+

*BFM, busulfan, fludarabine, melphalan; BU AUC, busulfan area under the curve; DSA, donor specific anti-HLA antibodies; n/d, not done; PT-CY, post-transplant cyclophosphamide; MMF, mycophenolate mofetil*.

Standard bacterial prophylaxis was used (Lefofloxacin) when ANC dropped below 0.5 × 10^9^/L. Antifungal prophylaxis with voriconazole was initiated following completion of BU administration. For *P. jirovecii* prevention, IV pentamidine was given until 1 year after BMT. Acyclovir was also started on admission for herpes simplex and varicella virus prophylaxis. Bi-weekly polymerase chain reaction (PCR) monitoring for cytomegalovirus (CMV) and weekly for adenovirus, Epstein–Barr virus (EBV), and human herpes virus-6 (HHV-6) were performed until discharge from hospital and subsequently at least every other week during the first 100 days and at least monthly for another 6 months.

Granulocyte-colony stimulating factor (G-CSF) was started on day +5 at 5 μg/kg/day until an absolute neutrophil count (ANC) of 2.5 × 10^9^/L was achieved for three consecutive days. Whole blood donor chimerism was evaluated on days +28, +100, +180, and +365. Since both patients had complete donor chimerism, lineage-specific chimerisms were not performed routinely but only at last follow-up, specifically at 27 months for patient #1 and at 12 months for patient #2 (Table 2).

**Table 2:** Post-transplant course.

Mucositis (grade)	III	II
Days to ANC >0.5 × 10^9^/L	16	13
Days to platelet >20 × 10^9^/L	36	22
Donor Chimerism d +28, +100, +180, +365 (%)	100	100
T-cell CD3^+^	100	100
Myeloid CD33^+^	100	100
Donor CMV serology	+	+
Recipient CMV serology	+	+
CMV reactivation	No	No
Adenovirus reactivation	No	No
HHV6 reactivation	No	No
EBV reactivation	No	No
Gram+ bacteremia	No	No
Gram+ bacteremia	Yes	Yes
Fungal infection	No	No
Acute GvHD	No	No
Chronic GvHD	No	No
Immune reconstitution 1 yr (/μl)
CD3^+^	1,759	1,200
CD4^+^	949	666
CD8^+^	752	399
CD4/CD8 ratio	1.3	1.7
CD19^+^	1,027	881
CD16^+^/CD56^+^	127	74
IVIG post-BMT (# doses)	0	1
EFS Post-BMT (mo)	28	12.5

*ANC, absolute neutrophil count; EFS, event free survival, lineage specific chimerisms were performed at 27 months and 12 months for patient #1 and #2, respectively*.

Patients and transplant characteristics are summarized in Table 1. Post-transplant courses were very similar with both siblings engrafting promptly with neither one developing signs of acute or chronic GvHD (Table 2). Except for mucositis, there were no other organ toxicities. No viral reactivations or disease occurred; however, both brothers developed stenotrophomonas maltophilia bacteremia at 4 and 5 months post-BMT, necessitating line removal and antibiotics (Table 2). Only the older brother required a single infusion of IVIG replacement therapy on day +30 post-haplo-BMT. Both siblings are alive and well with normal blood counts at 28 and 12.5 months post-transplant.

## Discussion

While haplo-HCT with PT-CY has been widely used for hematologic malignancies, this approach has only recently emerged for patients with WAS with only a handful of published reports. A study from Brazil described the application of haplo-BMT with PT-CY for primary immunodeficiencies, which included nine patients with WAS (13). Two of the three patients that received the Baltimore reduced intensity conditioning (RIC) (14) failed to engraft while all six of the patients receiving MAC with BU 16 mg/kg FLU 160 mg/m^2^ and rabbit ATG had stable engraftment and were alive. The incidence of GvHD was not reported specifically for the WAS patients, but they represented more than a third of a group that had 36% grade II–IV aGvHD and 17% cGvHD. Using BU 16 mg/kg and a slightly lower FLU dose (150 mg/m^2^) and rabbit ATG, Yue et al. reported five WAS patients from China (15). These patients received a combination of BM and peripheral blood stem cells (PBSCs) from the same donor resulting in no graft failures but with two patients developing cGvHD. Also using BU 16 mg/kg and FLU 150 mg/m^2^ but with alemtuzumab instead of ATG was another case series from Israel, which included a patient with WAS receiving haplo-BMT and engrafting without GvHD (16). A case report from Thailand used a lower dose of BU 320 mg/m^2^ but much higher dose of FLU 210 mg/m^2^ and rabbit ATG with engraftment and no GvHD (17). Other case series using different preparative regimens include one from India of primary immune deficiencies undergoing haplo-HCT with PT-CY that included five patients with WAS (18). Conditioning appeared to be RIC with FLU-MEL but the doses were not specified. Two of five patients developed graft failure and did not survive, each one having received BM and PBSCs. Of the three patients that engrafted, the two that received PBSCs developed grade II aGvHD and one cGVHD. A patient with WAS from India received PBSCs following myeloablative conditioning with thiotepa (TT) 7 mg/kg, BU 12.8 mg/kg, FLU 150 mg/m^2^, and rabbit ATG. He achieved engraftment without reported acute or chronic GvHD (19). Finally, in another case from India, rabbit ATG and thiotepa 10 mg/kg were added to the Baltimore regimen of CY, FLU, and TBI resulting in engraftment following PBSC haplo-transplant without acute or chronic GvHD (20). These reports are summarized in Table 3.

**Table 3:** T-cell replete haploidentical HCT reports with PT-CY in patients with Wiskott-Aldrich Syndrome.

	***n***	**Country**	**Intensity *n* =**	**Regimen**	**Graft *n* =**	**Engraft*n* (%)**	**aGvHD III-IV *n* (%)**	**cGvHD *n* (%)**
Fernandes et al. (13)	9	Brazil	RIC 3 MAC 6	CY-FLU-200cGy BU-FLU-ATG	BM	2 (67) 6 (100)	NR	NR
Yue et al. (15)	5	China	MAC	BU-FLU-ATG	BM + PBSC	5 (100)	0	2 (40)
Even-Or et al. (16)	1	Israel	MAC	BU-FLU-ALEMTUZ	BM	1 (100)	0	0
Kreetapirom et al. (17)	1	Thailand	MAC	BU-FLU-ATG	PBSC	1 (100)	0	0
Uppuluri et al. (18)	5	India	RIC 4 MAC 1	FLU-MEL FLU-TREO-200 cGy	PBSC 3, BM 2	3 (60)	0	1 (20)
Sharma et al. (19)	1	India	MAC	TT-BU-FLU-ATG	PBSC	1 (100)	0	0
Thakkar et al. (20)	1	India	RIC	TT-BU-FLU-ATG	PBSC	1 (100)	0	0
Katsanis, current	2	Tucson AZ, US	MAC	BU-FLU-MEL	BM	2 (100)	0	0

*PT-CY, post-transplant cyclophosphamide; n, number; aGvHD, acute graft vs. host disease; cGvHD, chronic graft vs. host disease; MAC, myeloablative conditioning; RIC, reduced intensity conditioning; CY, cyclophosphamide; FLU, fludarabine; BU, busulfan; ATG, anti-thymocyte globulin; ALEMTUZ, alemtuzumab; MEL, melphalan; TREO, treosulphan; TT, thiotepa; BM, bone marrow; PBSC, peripheral blood stem cells; NR, not reported*.

We present two brothers who underwent a T-cell replete haplo-BMT with PT-CY from their father. The siblings received MAC, which we have previously reported in haplo-BMT with PT-CY for hematologic malignancies and consists of BU-FLU-MEL but with the addition of rabbit ATG (21–23). Our preparative regimen has only 3 days of BU (12 mg/kg) and a lower dose of FLU (120 mg/m^2^) than the aforementioned regimens, as MEL is added to the last day of FLU. As indicated above, this regimen was well-tolerated with minimal organ toxicity limited to mucositis, trilineage engraftment with complete donor chimerism, no acute or chronic GvHD, no bacterial infections in the first 3 months post-BMT, and no fungal infections or viral reactivations. BU-based MAC conditioning regimens are more commonly used in WAS with MSD, MUD, MMUD, and UCB transplants and found to be associated with decreased graft failure and lower incidence of mixed chimerism when compared to RIC (2, 8–10). This also appears to be true with haplo-HCT as RIC regimens had higher numbers of patients with WAS developing graft failure (13, 18). While the numbers of patients with WAS reported to have received haplo-HCT is small, there appears to be a trend of more aGvHD and cGvHD in those receiving PBSCs compared to BM as has been documented in other transplant settings (24–26).

As WAS is an X-linked disorder, with an incidence of 1–4 per 1 million males, it would not be uncommon to have more than one boy in a family with this diagnosis. Our experience is unique as there have not been other reports of two brothers with WAS receiving haplo-BMT from the same donor, in this case their father. Moreover, it illustrates that haplo-BMT from the same donor, preferably the father, can be readily performed and be curative. While mothers, who are WAS carriers, have been used in some cases, haploidentical fathers or brothers are preferable. Additionally, the risk of GvHD is believed to be lower when male donors are used for male recipients compared to female donors. It is believed that female donor T cells may react to minor histocompatibility antigens encoded by genes present in the Y chromosome (27).

Our patients were of mixed race, their two sisters were not an HLA match, and unrelated searches failed to identify suitable matches as is the case more often than not for minority populations (28). Considering the difficulty in finding matched unrelated donors for minority patients, haplo-BMT with PT-CY has quickly become an acceptable alternative not only for hematologic malignancies but also non-malignant disorders such as WAS. Moreover, haploidentical familial donors, especially parents, are often eager to donate, dependable, and readily available for not only the initial BM donation but also additional collections as was the case with the father in our report. In summary, our cases, in addition to other recently published reports, indicate that MAC followed by haplo-HCT with PT-CY may be a safe and effective alternative to MUD and UCB transplantation for patients with WAS; however, it remains to be confirmed in a larger cohort of patients.

## Data Availability Statement

The raw data supporting the conclusions of this article will be made available by the authors, without undue reservation.

## Ethics Statement

The studies involving human participants were reviewed and approved by Human Subjects Protection Program The University of Arizona. Written informed consent to participate in this study was provided by the participants' legal guardian/next of kin.

## Author Contributions

EK wrote the manuscript and managed the patients during their hematopoietic cell transplantation. JS and JA reviewed the clinical records and co-wrote the manuscript. NR and PA edited the manuscript and treated the patients before transplantation.

## Conflict of Interest

The authors declare that the research was conducted in the absence of any commercial or financial relationships that could be construed as a potential conflict of interest.

## Publisher's Note

All claims expressed in this article are solely those of the authors and do not necessarily represent those of their affiliated organizations, or those of the publisher, the editors and the reviewers. Any product that may be evaluated in this article, or claim that may be made by its manufacturer, is not guaranteed or endorsed by the publisher.
